# Problems associated with alcohol consumption by university students

**DOI:** 10.1590/0104-1169.3579.2475

**Published:** 2014

**Authors:** Guillermo Alonso Castaño-Perez, Gustavo Adolfo Calderon-Vallejo

**Affiliations:** 1 PhD, Associate Professor, Escuela de Postgrados, Fundación Universitaria Luis Amigó, Medellín, Colombia; 2 MSc, Associate Professor, Facultad de Psicología y Ciencias Sociales, Fundación Universitaria Luis Amigó, Medellín, Colombia

**Keywords:** Alcohol Drinking, Students, Social Problems

## Abstract

**OBJECTIVES:**

the aim of this study was to analyze alcohol consumption by university students and psychosocial problems related.

**METHOD:**

descriptive correlational study that included 396 university students. The "Alcohol Use Disorders Identification Test" - (AUDIT) - and an "ad hoc" questionnaire were used as instruments to assess the associated problems.

**RESULTS:**

of the total sample, 88.6% drank, 20.5% had harmful consumption and 14.9% were at risk of dependence according to AUDIT. The study showed important results related to harmful alcohol consumption and dependence, with damage to the academic performance, social relationships, psychological status and sexual condition.

**CONCLUSIONS:**

complications caused by problematic alcohol consumption by university students, which is high in this group due to the high prevalence of their alcohol consumption, highlights the importance of promoting programs to prevent the abuse and dependence of this substance in universities.

## Introduction

The study of alcohol consumption by university students has generated interest in all cultures and all continents. Studies to explain the intake of this substance in this population began in the mid-seventies in industrialized countries^(^ 1 ^-^ 2 ^)^. From the eighties, there were reports on high alcohol consumption and problems associated with alcohol abuse in this population group^(^ 3 ^)^. Some authors report a greater likelihood of use of psychoactive substances through adolescence and early adulthood, with a positive association between educational level and consumption^(^ 4 ^)^. 

The problems resulting from alcohol consumption in young people are different from those in adults. In young people, the negative effects deriving from alcohol consumption often involve changes in the relationship with family, peers and teachers, poor school performance, aggression, crime, public disorder and high-risk behaviors, such as driving after drinking, as well as unprotected sexual activities, involving unintended pregnancy and sexually transmitted diseases^(^ 5 ^-^ 6 ^)^. In general, students who drink large amounts of alcohol have more risk behaviors for themselves and for the others compared to students who do not drink^(^ 7 ^)^.

In the same context, it has been argued that alcohol abuse and alcoholism participate directly and indirectly in the development of physical, mental and social damages^(^ 8 ^)^, besides affecting others and producing domestic violence, marital conflicts, economic problems, fights with injuries and traffic accidents^(^ 9 ^-^ 10 ^)^. All these problems not only affect the physical, emotional and social development of young people, but also their permanence at university and the quality of their training for working life. 

This research is focused on the analysis of the problems associated with the consumption of this substance according to the "Alcohol Use Disorders Identification Test" - AUDIT - and its correlation with problems in academic performance, relationships, family, psychological and sexual areas, in a sample of university students, thus contributing to the knowledge, because studies rarely address such associations, specifically in this population.

## Methods

This was a cross-sectional, descriptive correlational study, which investigated the problems associated with alcohol consumption in a sample of university students, according to the risks of the AUDIT scale.

### Sample

Stratified random sampling was used to calculate the sample size, considering the number of students enrolled in the first semester of 2010 at a university, in which the following parameters were considered: population size of 6,288 students; expected proportion of alcohol consumption of 88.0%, a confidence level of 95% and a design effect of 1.5. The sample size included in the study was 396 students of both sexes, aged between 15 and 49 years.

### Instruments

Questionnaire "ad hoc" to determine the socio-demographic characteristics and the difficulties associated with alcohol consumption: information collection on age; gender; marital status; socioeconomic status, assessed according to the classification proposed by the National Bureau of Statistics of Colombia; semester the student is enrolled in; alcohol consumption; age of first use; problems related to their academic performance; risks associated with their sexuality; physical aggressions; traffic accidents; problems with the authorities; changes in their physical or mental health, diagnosed by the university physician together with the clinical history and the clinical examination, among other characteristics, medical psychometric tests, the Zung test for depression and Hamilton test for anxiety; and family and social problems reported by the students themselves. This instrument was validated by three experts in drugs research (two psychiatrists and a sociologist) and through a pilot test applied to 30 individuals of the same university, college students who were not included in the final sample.

The "Alcohol Use Disorders Identification Test" - AUDIT- was developed by the World Health Organization (WHO), and approved in Colombia for the university population by Londoño^(^ 11 ^)^, resulting in Cronbach's alpha values of 0.86 and 0.90 in the test and re-test, respectively. The AUDIT includes questions about the consequences of alcohol consumption, along with questions about the amount and frequency of intake. It consists of 10 items, with 5 possible answers, scored from 0 to 4, except items 9 and 10, which score only 0, 2, 4. The result is obtained by the sum of the domain scores: risk-free consumption, dependence and hazardous consumption.

### Procedure for data collection

Approval was obtained from the directors of the university, who were previously informed on the project objectives. After authorization, the survey was applied in the same selected sample and each of the study participants signed an informed consent.

### Data analysis

The description of the results was based on descriptive statistical instruments and techniques, using SPSS 18.0, according to the variable type. In this context, frequency distributions for qualitative variables and summary central trend measures were performed for the quantitative variables, taking into consideration whether they followed a normal distribution by means of the Kolmogorov-Smirnov test with Lilliefors correction. 

After the descriptive process, the association between alcohol consumption and other variables included in the form were estimated by chi-square and Student's t-test in terms of their applicability and considering for statistically significant associations p ≤ 0.05.

## Results

Women represented 60.1% of the sample, the mean age was 23.2 years (SD=± 5.7) and 75% of the sample was between 18 and 25 years, mostly single (81.6%) and belonging to the socio-economic groups 3 and 4 (74.7%) (Table 1).

**Table 1:** - Socio-demographic data. Alcohol consumption and related problems in university students. Medellin, Colombia, 2010 (N=396)

Variable		Results N(%)
Age*		
	Under 18 years old	(13) 3.3%
	18-25 years old	(297) 75%
	Over 25 years	(86) 21.7%
Gender		
	Female	(238) 60.1%
	Male	(158) 39.9%
Marital status		
	Single	(323) 81.6%
	Married	(38) 9.6%
	Engaged	(27) 6.8%
	Separated	(8) 2%
Socio-economic level^†^		
	Section 1 e 2	(71) 18%
	Section 3	(198) 50%
	Section 4	(98) 24.7%
	Section 5 e 6	(27) 6.8%

* Mean age: 23.2 years (SD=5.7)

† In Colombia, the socio-economic stratification occurs from 1 to 6, with 1 being the lowest and 6 the highest. This classification is made by the National Administrative Department of Statistics - NADS - according to the type of residence and all the amenities it contains.

With regard to alcohol consumption, 88.6% (351) of the respondents reported alcohol consumption across the lifetime. The mean age of onset of alcohol consumption was 14.4 years (SD=± 3.2), and 11.3% did so before age 10 and 58.7% had experienced it between age 11 and 15 years old.

According to the AUDIT score, 64.6% had no problems with alcohol, while 20.5% showed hazardous drinking and 14.9% were at risk of dependence. 

Regarding the presence of problems related to the academic performance and the Alcohol Use Disorders Identification Test - AUDIT-, statistically significant associations were found, which shows that, the greater the problem of hazardous consumption or dependence, the greater the significance (p = 0.000). Among the problems presented in the cases of abuse or addiction to psychoactive substances, the absences and delays in the classes are highlighted (37.3% if there is hazardous consumption and 67.4% if there is dependence) and low academic performance (16.4% in the consumption abuse and 52.2% if there is dependence (Table 2).

**Table 2:** - Academic problems and their correlation with the AUDIT. Alcohol consumption and related problems in university students. Medellin, Colombia, 2010 (N=351)

Academic problems and AUDIT		Yes			No		Total	P value
		n	%		n	%		
Absences or delays in the classes								≤0.001
	Risk-free consumption	24	12.3		171	87.7	195	
	Hazardous consumption	41	37.3		69	62.7	110	
	Dependence	31	67.4		15	32.6	46	
Low academic performance								≤0.001
	Risk-free consumption	5	2.6		190	97.4	195	
	Hazardous consumption	18	16.4		92	83.6	110	
	Dependence	24	52.2		22	47.8	46	

Statistical significance was also found by relating problems in personal, sexual and physical health aspects with drinking practices, according to the AUDIT classification.

Sexually Transmitted Diseases were more frequent in people with risk drinking (3.6%) and dependence (8.7%) according to the AUDIT, with a statistical association and p ≤ 0.005. A statistical association between AUDIT and unintended pregnancy was also observed in this sample (p=0.02). Among those presenting risk drinking, 7.3% had unintended parenthood, increasing the percentage to 13.0% in those who scored at risk of dependence. General health problems (headache, joint pain, gastrointestinal disorders) are also more frequent among those who score at risk for dependence (41.3%) or hazardous consumption (18.2%), with statistically significant results (p = 0.000). 

Physical aggressions are also more common among those with dependence (21.7%), as well as traffic accidents (13.0%) and problems with the authorities (23.9%) (Table 3).

**Table 3:** - Personal problems and their correlation with the AUDIT. Alcohol consumption and related problems in university students. Medellin, Colombia, 2010 (N=351)

Personal problems and AUDIT		Yes			No		Total	P value
		n	%		n	%		
Infection with STDs								0.005
	Risk-free consumption	1	0.5		194	99.5	195	
	Hazardous consumption	4	3.6		106	96.4	110	
	Dependence	4	8.7		42	91.3	46	
Unwanted parenting								0.022
	Risk-free consumption	6	3.1		189	96.9	195	
	Hazardous consumption	8	7.3		102	92.7	110	
	Dependence	6	13.0		40	87.0	46	
Physical aggressions								0.000
	Risk-free consumption	5	2.6		190	97.4	195	
	Hazardous consumption	6	5.5		104	94.5	110	
	Dependence	10	21.7		36	78.3	46	
Traffic accident as a driver or pedestrian								0.022
	Risk-free consumption	6	3.1		189	96.9	195	
	Hazardous consumption	8	7.3		102	92.7	110	
	Dependence	6	13.0		40	87.0	46	
Problems with the authorities for driving under the influence of alcohol								0.000
	Risk-free consumption	3	1.5		192	98.5	195	
	Hazardous consumption	6	5.5		104	94.5	110	
	Dependence	11	23.9		35	76.1	46	
Health problems								0.000
	Risk-free consumption	8	4.1		187	95.9	195	
	Hazardous consumption	20	18.2		90	81.8	110	
	Dependence	19	41.3		27	58.7	46	

When analyzing the correlation between the AUDIT classification and alcohol consumption effects on social and familial relationships, significant associations were observed between fights and arguments with friends (p ≤ 0.001), fights and arguments with strangers (p ≤ 0.001), loss of friends (p = 0.009), loss of partners (p ≤ 0.001) and conflicts with parents (p ≤ 0.001). Fights and arguments with friends are the most frequent problems (25.5% in case of abusive consumption and 52.2% if there is dependence); followed by conflicts with parents (25.5% if there is consumption of risk and 50% if there is dependence), fights and arguments with strangers (14.5% and 41.3% if abuse occurs, if dependence occurs) and a small percentage of loss of partners and friends (Table 4).

**Table 4:** - Social and family problems and their correlation with the AUDIT. Alcohol consumption and related problems in university students. Medellin, Colombia, 2010 (N= 351)

Social and familial problems and AUDIT		Yes			No		Total	Value p
		n	%		n	%		
Fights and arguments with friends								≤0.001
	Risk-free consumption	26	13.3		169	86.7	195	
	Hazardous consumption	28	25.5		82	74.5	110	
	Dependence	24	52.2		22	47.8	46	
Fights and arguments with strangers								≤0.001
	Risk-free consumption	12	6.2		183	93.8	195	
	Hazardous consumption	16	14.5		94	85.5	110	
	Dependence	19	41.3		27	58.7	46	
Loss of friends								0.030
	Risk-free consumption	11	5.6		184	94.4	195	
	Hazardous consumption	11	10.0		99	90.0	110	
	Dependence	8	17.4		38	82.6	46	
Loss of partners								≤0.001
	Risk-free consumption	12	6.2		183	93.8	195	
	Hazardous consumption	14	12.7		96	87.3	110	
	Dependence	18	39.1		28	60.9	46	
Conflict with parents								≤0.001
	Risk-free consumption	19	9.7		176	9.3	195	
	Hazardous consumption	28	25.5		82	74.5	110	
	Dependence	23	50.0		23	50.0	46	

Last but not least are the psychological problems among consumers who have difficulties with alcohol consumption according to the AUDIT. Statistical significance (p ≤ 0.001) was observed for each of the variables considered: depression (some degree, according to the test Zung), anxiety (some degree according to Hamilton's test), emotional oscillations (dysthymia), irritability and low tolerance. The major problems occurring in this area are the emotional oscillations, 50.0% if the consumption is rated according to the AUDIT as hazardous and 56.5%, if what occurs is dependence. Then comes anxiety (37.3% in cases of abusive consumption and 50.0% for dependence), depression (34.5% if there is hazardous consumption and 47.8% if dependence occurs) and irritability and low tolerance (21.8% in case of hazardous consumption and 37% for dependence) (Table 5).

**Table 5:** - Psychological problems and their correlation with the AUDIT. Alcohol consumption and related problems in university students. Medellin, Colombia, 2010 (N=351)

Psychological problems and AUDIT		Yes			No		Total	P value
		n	%		n	%		
Depression								≤0.001
	Risk-free consumption	19	9.7		176	90.3	195	
	Hazardous consumption	38	34.5		72	65.5	110	
	Dependence	22	47.8		24	52.2	46	
Anxiety								≤0.001
	Risk-free consumption	17	8.7		178	91.3	195	
	Hazardous consumption	41	37.3		69	62.7	110	
	Dependence	23	50.0		23	50.0	46	
Emocional Oscillations								≤0.001
	Risk-free consumption	38	19.5		157	80.5	195	
	Hazardous consumption	55	50.0		55	50.0	110	
	Dependence	26	56.5		20	43.5	46	
Irritability. low tolerance								≤0.001
	Risk-free consumption	17	8.7		178	91.3	195	
	Hazardous consumption	24	21.8		86	78.2	110	
	Dependence	17	37.0		29	63.0	46	

## Discussion

With regard to alcohol consumption by university students, there are many epidemiological reports conducted in different parts of the world, which differ according to the contexts and cultures. In Canada, in a sample of 6,282 university students, it was observed that alcohol is the most commonly used drug, with a prevalence of 77% in the past 30 days^(^ 12 ^)^. In the United States and Mexico, the university students generally report high rates of excessive drinking, abuse and dependence^(^ 13 ^)^. 

Among university students in the Andean region^(^ 14 ^)^, alcohol consumption is also high. Over 90% of students in Colombia and Peru, and about 75% in Bolivia and Ecuador, reported alcohol consumption at least once in their lives, values very similar to those found in our study (88.6%).

Another finding showing some similarities with our research results is related to alcohol consumption and the classification of the problem according to AUDIT. In the study of the Community of Andean Nations^(^ 14 ^)^ on consumers of alcohol in the past year, about a third part of the students in Bolivia, Colombia, Ecuador, and 21% in Peru are self classified as consumers of risk or hazardous alcohol consumers. In addition, by evaluating the signs of addiction, it is observed that almost 8% of university students in Peru, Bolivia 10.5%, 12% in Colombia and 16% in Ecuador, who claim to have consumed alcohol in the last year, can be considered with signs of addiction. In our study, 20.5% had risk drinking and 14.9% were at risk of dependence.

The university population is highly vulnerable to alcohol consumption^(^ 15 ^-^ 16 ^)^ due to the ease of buying alcoholic beverages, the availability of consumption situations that occurs by entering the university environment, the independence and autonomy they acquire and on many occasions, the lack of parental control.

In relation to the problems associated with alcohol consumption and their correlation with the AUDIT, no studies were found. Some studies that have been conducted, although very scarce, report the abuse of this substance and the problems caused in university students. It has been reported a higher default and dropout in this population group^(^ 17 ^-^ 18 ^)^. School dissatisfaction, higher amount of repetitions in the courses^(^ 19 ^)^ and larger family and social difficulties^(^ 20 ^-^ 22 ^)^ were also found. These results are similar to those found in our study.

Regarding the general population and students, there are several studies that report on the consequences of the consumption/abuse of this substance in all areas of the individual and a assembly is noted. Although these studies have been conducted in different populations, there are similarities when analyzing the problems caused by alcohol consumption and their impact on the student at work, in family, social relations, accidents, sexual and psychological state, which are probably higher among university students due to higher alcohol consumption in this group. Some results are described below without comparison with our results, because they are not comparable.

The associations between alcohol abuse with increased morbidity, violence, family problems, school dropout and accidents have been reported in different populations^(^ 23 ^)^. The World Health Organization revealed that in 2000, alcohol was responsible for 4.0% of the global burden of disease related to neuropsychiatric disorders (addiction, psychosis, depression) and unintentional injuries (accidents, burns, drowning and falls)^(^ 24 ^)^ and some authors have called attention to the damage that alcohol abuse causes to health, increasing gastrointestinal and cardiovascular diseases, traffic accidents, violent deaths and the spread of sexually transmitted diseases due to excessive use of this substance and unprotected sexual practices^(^ 25 ^-^ 26 ^)^.

On the other hand, family and social conflicts are other consequences of alcohol abuse among young people. Rejection and isolation occurs as consequence of excessive alcohol consumption (negative prognosis from family, friends and society) and are typical features of this kind of problem^(^ 27 ^)^. Our study also showed association between these problems and hazardous consumption or dependence according to the AUDIT.

## Conclusion

The high prevalence and the associations with the identified problems emphasize the need to create practical interventions within the Universities and to develop public policies for health promotion and disease prevention, such as early pregnancy, mental problems, traffic accidents and many others.

The observations in this study are consistent with those empirically demonstrated by other authors, both in university as students, and reinforce the importance to assess the level of alcohol consumption and damages related to risk drinking and dependence in young people. Therefore, it is necessary to develop effective, efficient and productive prevention strategies and to educate about the drinking limits more than just banning the alcohol consumption. Desiringa sober society in the West is and will always be a utopia, but not when it comes to intervening in university students, whose stages of life include alcohol as part of their social development.

## References

[1] Alvira- Martin F (1982). Pautas de consumo de bebidas alcohólicas entre los jóvenes
Españoles.

[2] Brook M, Walfish S, Stenmark D, Canger J (1981). Personality variables in alcohol abuse in college
students. J Drug Educ..

[3] O' Conell D, Patterson H.A (1989). Survey of current college alcohol abuse programs,
attitudes and training needs. J Alcohol Drug Educ..

[4] Velásquez J, Scoppetta O (1998). Consumo de sustancias psicoactivas en estudiantes de carreras
técnicas y tecnológicas de Santa Fe de Bogotá.

[5] American Academy of Pediatrics (1995). Uso y abuso del alcohol: una preocupación
pediátrica. Pediatrics (ed. esp.).

[6] Stueve A, O´Donnell LN (2005). Early alcohol initation and subsequent sexual and
alcohol risk behaviours among urban youths. Am J Public Health..

[7] Hingson RW, Heeren T, Winter MR (2006). Age at drinking onset and alcohol dependence: Age at
onset, duration, and severity. Arch Pediatr Adolesc Med..

[8] Gutiahr E, Gmel G, Rehm J (2001). Relation betwin average alcohol consumption and disease:
an overview. Eur Addict Res..

[9] Tempier R, Boyer R, Lambert J (2006). Psychological distrress among female spouses of male at
- risk drinkers. Alcohol..

[10] MacDonald S, Cherpitel CJ, DeSouza A, Stockwell T, Borges G, Giesbrecht N (2006). Variations of alcohol impairment indifferent types,
causes and contexts of injuries: Results de emergency room studies from 16
countries. Accid Anal Prev..

[11] Londoño C (2004). Construcción de un modelo cognitivo-social integrado para la
prevención del abuso en el consumo de alcohol en universitarios bogotanos.

[12] Hingson RW, Heeren T, Winter MR (2006). Age at Drinking Onset and Alcohol
Dependence. Arch Pediatr Adolesc Med..

[13] Johnston LD, O'Malley PM, Bachman JG, Schulenberg JE (2007). Monitoring the Future national survey results on drug use,
1975-2004: Volume II.

[14] SG-CAN, Unión Europea (2009). Estudio Epidemiológico Andino sobreconsumo de drogas sintéticas
en la población universitaria de Bolivia, Colombia, Ecuador y Perú
[Internet].

[15] Flórez L (2007). Diagnóstico e Intervención del Consumo Excesivo de
Alcohol en Ambientes Educativos. TIPICA: Boletín Electrónico de Salud Escolar. [Internet].

[16] Palma M, Lannini J, Moreno S (2005). Validación de la Prueba Young Adult Alcohol Problems
Screening Test, YAAPST, en un grupo de estudiantes universitarios de la Pontificia
Universidad Javeriana de Bogotá. Universitas Psychol..

[17] Londoño C, García W, Valencia S, Vinaccia S (2005). Expectativas frente al consumo de alcohol en jóvenes
universitarios colombianos. Rev Anales Psicol..

[18] Crum RM, Ensminger ME, Ro MJ, McCord J (1998). The association of educational achievement and school
dropout with risk of alcoholism: A twenty - five year prospective study of
inner-city children. J Stud Alcohol..

[19] Wichstrom L (1998). Alcohol intoxication and school dropout. Drug Alcohol Rev..

[20] López-Frías M, Fernández MF, Planells E, Miranda MT, Mataix J, Llopis J (2001). Alcohol consumption and school efficiency in Spanish
secondary school students. J Stud Alcohol..

[21] Donovan J (2004). Adolescent Alcohol Initiation: A Review of Psychosocial
Risk Factors. J Adolesc Health..

[22] Herrera Paredes JM, Arena Ventura CA (2010). Alcohol consumption and domestic violence against women:
a study with university students from Mexico. Rev. Latino-Am. Enfermagem..

[23] Reinaldo AMS, Pillon SC (2008). Alcohol effects on family relations: a case
study. Rev. Latino-Am. Enfermagem..

[24] Plan Nacional sobre Drogas (2007). Encuesta domiciliaria sobre alcohol y drogas en España
(EDADES), 1995-2007 [Internet].

[25] Organização Mundial da Saúde. OMS (2005). Alcohol, Gender and Drinking Problems. Perspectives from Low
and Middle Income Countries. - GENASIS- OPS- Edited by Isidore S. Obot y Robin
Room.

[26] Perez A (2009). Transiciones en el consumo de drogas en
Colombia. Adicciones..

[27] Calafat Far A, Juan Jerez M, Duch Moyá MA (2011). Conductas de riesgo de jóvenes turistas españoles de
vacaciones en Mallorca e Ibiza: consumo de alcohol, drogas y otros riesgos para la
salud. Rev Española Drogodependencias..

